# Statistics on steroids: How recognizing competing risks gets us closer to the truth about COVID-19-associated VAP

**DOI:** 10.1186/s13054-022-04264-x

**Published:** 2022-12-21

**Authors:** Andrew F. Shorr, Marya D. Zilberberg

**Affiliations:** 1 Medstar Washington Hospital Center (AFS), Room 2a69D, 110 Irving St., NW, Washington, DC 20010 USA; 2 EviMed Research Group. LLC (MDZ), Goshen, MA USA

Corticosteroids represent a major tool for the treatment and management of infection due to severe to acute respiratory syndrome coronavirus 2 (SARS-CoV-2). Multiple large platform trials have demonstrated the value of corticosteroids for the care of those suffering respiratory failure as a consequence of SARS-CoV-2 [1, 2]. When reported, clinicians greeted these studies with emotions ranging from relief to jubilation—as they showed that tools now existed for combating the virus and for addressing the high mortality risk accompanying severe infection [1, 2]. This was particularly true for intensivists who had seen many succumb to acute respiratory distress syndrome (ARDS). Because the data are so definitive, multiple national and international guidelines now recommend corticosteroids as part of the routine care for those sick enough with SARS-CoV-2 to merit them [3, 4]. The fact that few interventions subsequently studied have proven effective underscores the power of corticosteroids in the care of severe SARS-CoV-2.

In addition to the high mortality rate associated with SARS-CoV-2 in the intensive care unit (ICU), the pandemic has resulted in an increase in the rate of ventilator-associated pneumonia (VAP) [5, 6]. For many years, rates of VAP were falling due to the adoption of a multitude of interventions and a dedicated focus on prevention. Even after adjusting for the prolonged duration of mechanical ventilation (MV) required for SARS-CoV-2 respiratory failure, the incidence of VAP has risen at an alarming rate [5, 6]. This has led to a broader use of antibiotics and accelerated the already rising prevalence of infections due to multi-drug-resistant (MDR) bacterial pathogens [7]. VAP following SARS-CoV-2 also clearly adds to the risk of death in patients in whom we often have invested substantial resources.

Several obvious questions arise. Has the mortality benefit related to corticosteroids come at a price? Is there some nexus between corticosteroid use and VAP? The original trials examining corticosteroids did not collect information on rates of VAP so we cannot look to them to inform this debate. Fortunately, several more recent reports, all in *Critical Care*, have explored this crucial issue.

Scaravilli et al. investigated the connection between corticosteroid exposure and VAP in a multicenter cohort of 739 patients [8]. Utilizing a propensity score matching approach, these investigators concluded that early corticosteroid treatment significantly amplified the chance for VAP—nearly doubling it (hazard ratio: 1.81 (1.31–2.50), *p* = 0.0003) [8]. Similarly, Lamouche-Wilquin and co-workers determined that corticosteroids for SARS-CoV-2 led to a greater risk for VAP [9]. In their population of 670 subjects across multiple ICUs, the relationship between corticosteroids and VAP was less strong that that noted by Scaravilli et al. [8, 9]. Specifically, Lamouche‑Wilquin estimated that corticosteroids increased the risk of VAP by approximately 30% [9]. Although the diagnosis of VAP can prove challenging, both analyses relied on accepted definitions for VAP and required quantitative cultures to confirm the presence of acute bacterial infection [8, 9]. Moreover, sites in each report employed multiple VAP preventive strategies. Taken together, these observations deriving from the experience of nearly 30 European ICUs would seem to indicate that, although corticosteroids may be lifesaving in SARS-CoV-2-associated respiratory failure, they expose the patient to an elevated chance for VAP—and in turn the risk of an MDR infection and a longer duration of MV [8, 9].

A third report in *Critical Care*, however, questions this conclusion. Saura and colleagues scrutinized the impact of corticosteroids on VAP during the period before routine use of these agents [10]. Unlike other investigators, they ascertained that corticosteroid treatment did not lead to higher rates of VAP. Why the difference? The key lies in analytical paradigms. In contrast to other sets of authors, Saura et al. noted that the impact of corticosteroids on VAP varied based on the duration of MV [8–10]. In other words, they made the crucial observation that one could not assume that the risk for VAP as a function of corticosteroid treatment was constant over the entire duration of MV [10]. For example, a patient who does not receive corticosteroids and dies early cannot be at further risk for VAP. The risk for VAP, simply put, competes with an ongoing risk for death. Additionally, survival over time selects for a unique set of patients who, by virtue of some still unidentified intrinsic “sturdiness,” may not face the same chance for VAP as others. This fact is confirmed by their finding that the relationship between corticosteroids and VAP shifted over time [10].

The conclusions of Saura et al. should serve to reassure clinicians that if corticosteroid use comes with a cost in terms of VAP, it is, at most, a small cost [10]. Similarly, clinicians should not leap to believe that patients on corticosteroids for SARS-CoV-2 reflexively merit antibiotics for VAP if they decompensate—rather they should utilize rigorous diagnostic techniques to confirm VAP before embarking on a protracted course of treatment. Finally, intensivists must remain vigilant in their efforts to prevent VAP. One cannot blame the increased incidence of VAP during the pandemic purely on prolonged durations of MV or corticosteroids. Rather, we need to harken to and embrace the evidence-based interventions shown to reduce VAP while emphasizing other infection control practices that broke down during the early days of 2020.

## Data Availability

Not applicable.

## References

[1] Angus DC, Derde L, Al-Beidh F (2020). Effect of hydrocortisone on mortality and organ support in patients with severe COVID-19: the REMAP-CAP COVID-19 corticosteroid domain randomized clinical trial. JAMA.

[2] Dequin PF, Heming N, Meziani F (2020). Effect of hydrocortisone on 21-day mortality or respiratory support among critically Ill patients with COVID-19: a randomized clinical trial. JAMA.

[3] https://www.covid19treatmentguidelines.nih.gov/management/critical-care-for-adults/. Accessed 22 Sept 2022.

[4] https://www.sccm.org/SurvivingSepsisCampaign/Guidelines/COVID-19. Accessed 22 Sept 2022.

[5] Pickens CO, Gao CA, Cuttica MJ (2021). Bacterial superinfection pneumonia in patients mechanically ventilated for COVID-19 pneumonia. Am J Respir Crit Care Med.

[6] Ippolito M, Misseri G, Catalisano G (2021). Ventilator-associated pneumonia in patients with COVID-19: a systematic review and meta-analysis. Antibiotics (Basel).

[7] Kariyawasam RM, Julien DA, Jelinski DC (2022). Antimicrobial resistance (AMR) in COVID-19 patients: a systematic review and meta-analysis (November 2019–June 2021). Antimicrob Resist Infect Control.

[8] Scaravilli V, Guzzardella A, Madotto F (2022). Impact of dexamethasone on the incidence of ventilator-associated pneumonia in mechanically ventilated COVID-19 patients: a propensity-matched cohort study. Crit Care.

[9] Lamouche-Wilquin P, Souchard J, Pere M (2022). Early steroids and ventilator-associated pneumonia in COVID-19-related ARDS. Crit Care.

[10] Saura O, Rouze A, Martin-Loeches I (2022). Relationship between corticosteroid use and incidence of ventilator-associated pneumonia in COVID-19 patients: a retrospective multicenter study. Crit Care.

